# Atrial Fibrillation and Great Saphenous Vein Insufficiency: Exploration of Common Pathways in Hemodynamic Derangements and Systemic Inflammation

**DOI:** 10.31083/RCM48578

**Published:** 2026-07-20

**Authors:** Chang Wei, Ziqiang Sun

**Affiliations:** ^1^Department of Vascular Surgery, Affiliated Hospital of Jining Medical University, Jining Medical University, 272067 Jining, Shandong, China; ^2^Department of Vascular Surgery, Affiliated Hospital of Jining Medical University, 272067 Jining, Shandong, China

**Keywords:** atrial fibrillation, varicose veins, angiotensin

## Abstract

Atrial fibrillation is the most prevalent type of arrhythmia globally, the incidence of atrial fibrillation continues to rise. Concurrently, as lifestyles modernize, the prevalence of saphenous varicose veins is also increasing significantly. Although both conditions have received considerable clinical attention, previous studies have primarily focused on independent investigations within their respective fields, leaving the potential association between these conditions relatively unexplored. In recent years, retrospective clinical analyses have shown that saphenous varicose veins may constitute a novel risk factor for the onset of atrial fibrillation—a breakthrough discovery that has opened a new dimension of cross-border dialogue in investigating disease mechanisms. Therefore, this study aimed to develop an innovative framework for the pathophysiological association between atrial fibrillation and saphenous varicose veins and to elucidate the associated underlying mechanisms from a multidimensional, evidence-based medical perspective. Available evidence suggests that hemodynamic changes in the lower extremity veins can directly affect right heart preload via central venous pressure transmission, thereby inducing atrial electrophysiological remodeling. Activation of the RAAS (renin-angiotensin-aldosterone system) is involved in venous wall remodeling and in the activation of inflammatory cytokine networks that promote atrial fibrosis. Autonomic dysfunction also exhibits shared pathological features in venous insufficiency and the neural remodeling associated with atrial fibrillation. Moreover, the bidirectional alterations in blood flow shear force may not only accelerate venous valve injury but also trigger abnormal electrical activity in atrial myocytes. The pathophysiological bridging hypothesis proposed in this study not only challenges the traditional single-system research paradigm, but also provides important implications for clinical intervention strategies. Indeed, by integrating a multidisciplinary chain of evidence, this paper systematically demonstrates the potential impact of peripheral venous lesions on cardiac electrophysiological activity, thereby adding a new dimension to screening and identifying a potential therapeutic target for the prevention of atrial fibrillation. These findings both deepen understanding of the nature of the two diseases and lay a theoretical foundation for establishing an integrated cardiovascular-peripheral vascular diagnostic and treatment model.

## 1. Introduction

Atrial fibrillation (AF) is a complex condition resulting from various underlying pathophysiological abnormalities and is the most prevalent type of arrhythmia worldwide. Epidemiological studies indicate that AF affects approximately 1% of the general population, with incidence rates increasing exponentially with age; the prevalence rises to 10% among individuals over 65 years old [1]. According to 2020 epidemiological survey data from the United States, there are over 3.3 million confirmed cases of atrial fibrillation [2]. In contrast, the prevalence of atrial fibrillation among Chinese adults is 1.6% [3]. Furthermore, the risk of all-cause mortality in this patient population has significantly increased, with a 5-year mortality rate of 42.7% [4,5]. Given the acceleration of global ageing, the number of patients with atrial fibrillation is projected to exceed 12 million by 2030, underscoring the urgent need for the development of preventive intervention strategies [6]. Varicose veins are a common manifestation of degenerative lesions in the venous system, characterised by the tortuous dilation of superficial veins in the lower extremities, often accompanied by vascular wall injury, inflammatory effusion, and thrombotic complications [7]. The incidence of varicose veins significantly increases in the elderly population, with a global prevalence of nearly 30% [8]. The occurrence and progression of this condition are notably comorbid with cardiovascular risk factors such as obesity, smoking, hypertension, and physical inactivity [9]. Previous studies have primarily focused on the association between atrial fibrillation and arterial thromboembolism, while the potential link between atrial fibrillation and venous system diseases has been rarely explored [10,11]. Notably, the FinnGen has identified a genetic causal association between varicose veins and atrial fibrillation through two-way Mendelian randomisation (MR) analysis. The inverse variance-weighted model (IVW) demonstrated that each standard deviation increase in the genetic risk score for varicose veins is associated with a 6% increase in the risk of atrial fibrillation (OR = 1.06, 95% CI 1.03–1.10, *p* = 0.001) [9,12]. This study focuses on the pathophysiological interaction network between atrial fibrillation and varicose veins, aiming to elucidate the biological basis of the causal association between the two diseases by systematically examining the core mechanisms involving inflammatory mediators, endothelial function, and biomechanical changes. Further exploration of targeted venous function improvement and anti-inflammatory intervention strategies holds significant translational value for the preventive management of high-risk groups for atrial fibrillation and provides an evidence-based medical foundation for establishing atrial fibrillation screening standards for patients with varicose veins. Reason: Improved clarity, readability, and technical accuracy while correcting grammatical and punctuation errors.

## 2. Clinically Relevant Evidence

A retrospective study utilising the Taiwan National Health Insurance database found that the incidence of atrial fibrillation in cohorts with varicose veins was 4.82 per 1000 person-years, compared to 3.47 per 1000 person-years in the control cohort. After adjusting for all confounding variables, individuals with varicose veins exhibited a significantly increased risk of developing atrial fibrillation compared to the control group (HR = 1.23, 95% CI: 1.04–1.45), demonstrating for the first time that varicose veins are associated with atrial fibrillation [13]. In another observational study, 392 patients with atrial fibrillation were divided into two groups: 218 (56%) with chronic atrial fibrillation and 174 with non-chronic atrial fibrillation. Results: Doppler ultrasound showed that the venous return rate of patients with chronic atrial fibrillation was higher (38% vs. 16%, *p* < 0.05). There was a correlation between the duration of atrial fibrillation, right atrial volume index, and CEAP (Clinical, Etiologic, Anatomic, Pathophysiologic) grade (RHO (Spearman’s Rank Correlation Coefficient (ρ)): 0.314, *p* < 0.001) and (RHO: 0.258, *p* < 0.001), respectively [14]. A recent cohort study based on the Korean population found a potential association between varicose veins (VV) and an increased risk of developing AF, with 24,557 (0.91%) having VV, including 3684 (0.14%) severe VV and 20,873 (0.77%) mild VV. During a median follow-up of 10.06 years, 24,557 (0.92%) cases of AF occurred. After pre-match (HR: 1.13, 95% CI: 1.06–1.21, *p* < 0.001) and 1:3 propensity score matches (PSM) (HR: 1.17, 95% CI: 1.08–1.27, *p* < 0.001), participants with VV demonstrated a higher risk of AF incidence [15]. In addition, a 78-year-old male patient who underwent varicose vein stripping and ligation of his right leg developed epigastric pain on the second day after surgery, with a new onset of AF observed on an electrocardiogram (ECG) [16]. Therefore, further research is needed on whether variceal ligation and dissection lead to a higher complication rate of postoperative atrial fibrillation than minimally invasive procedures such as sclerotherapy or laser ablation and whether improved venous return therapy has a potential preventive effect on atrial fibrillation.

## 3. Pathophysiological Bridge

### 3.1 Mechanical-Electrical Remodeling Effects of Central Venous Pressure Conduction

**Fig. 1.**
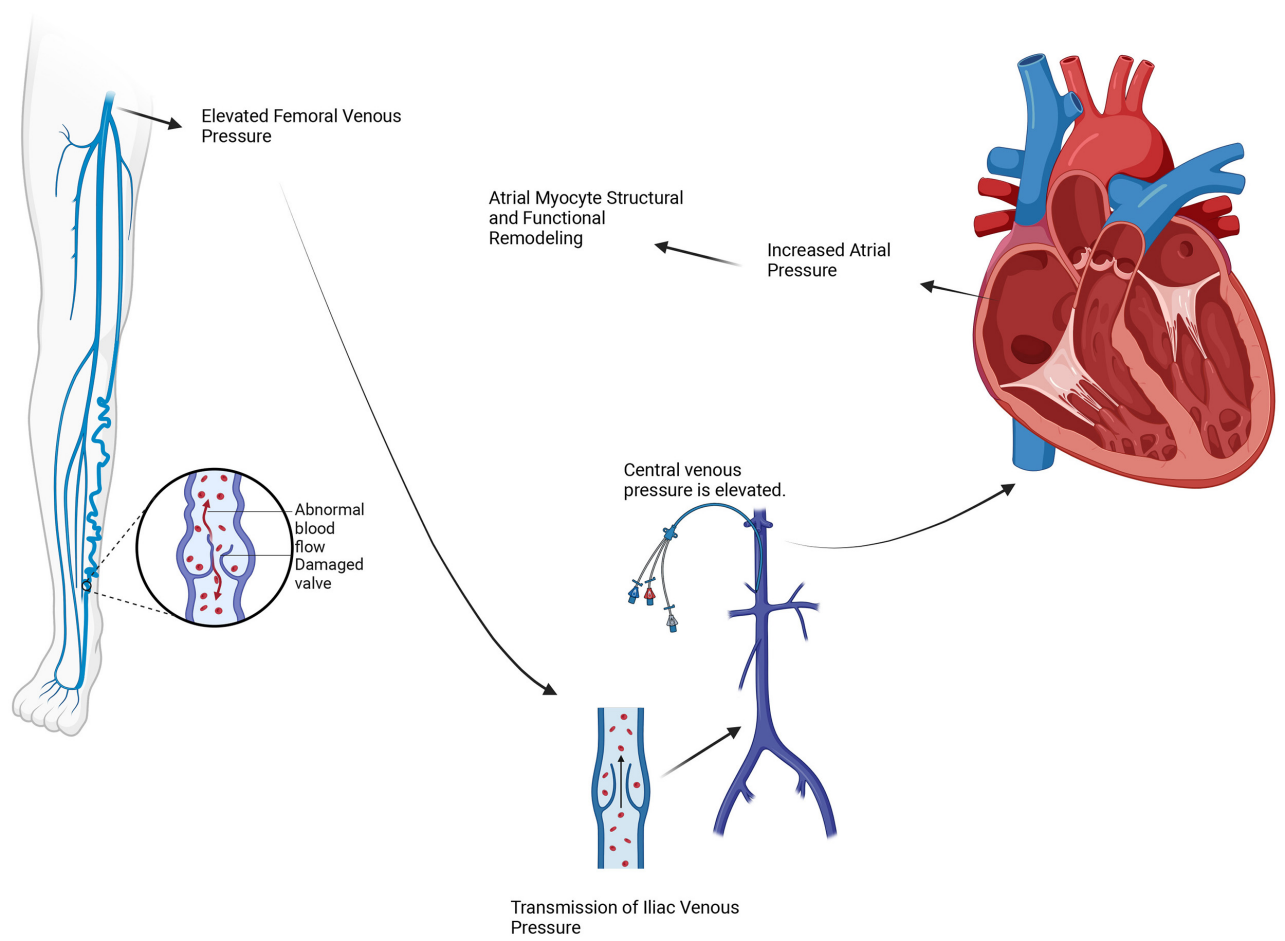
**Mechanical–electrical remodelling effect of central venous pressure transmission**. The figure was created using BioRender.

Saphenous vein valve insufficiency, which serves as the primary pathological basis for superficial lower extremity vein disease, initiates cardiac electrophysiological remodelling through multi-level haemodynamic disturbances and constitutes a significant peripheral contributor to the development of atrial fibrillation [17,18]. Perforating vein insufficiency is common among patients with lower extremity varices, with a study indicating that the prevalence of perforating vein insufficiency in patients with severe varices reached as high as 58.3 per cent when evaluating the correlation between dysfunctional perforating veins and vein pigmentation [19]. Another study assessing the relationship between the clinical classification of chronic venous insufficiency and duplex ultrasound findings revealed a 30.8 per cent incidence of deep venous reflux and a higher rate of perforating branch insufficiency in patients with severe varices [20]. Normal perforated vein flow has been found to be unidirectional (from superficial to deep veins), but increased cutaneous blood flow during muscle contraction (eg, exercise) significantly enhances spontaneous flow to perforating veins [21]. Therefore, we believe that the presence of perforating venous insufficiency will lead to an abnormal increase in blood volume in the deep veins, and when the muscles are contracted, the muscle pump cannot effectively empty the blood, resulting in an increase in deep vein pressure, which may also cause secondary damage to the deep vein valves, forming a vicious circle. Specifically, structural abnormalities of the valves result in a dynamic pressure gradient between the femoral vein and the non-functioning venous segment, resulting in a significant increase in femoral venous pressure in response to a calf-muscle pump [22,23]. Changes in femoral vein haemodynamics (e.g., increased flow or change in resistance) can affect pressure in the proximal iliac vein [24]. Elevated iliac venous pressure is transmitted to the right atrium through the inferior vena cava (IVC), and the diameter of the IVC as a volume vessel fluctuates with central venous pressure (CVP) (CVP is positively correlated with IVC diameter, r = 0.82, *p* < 0.01) [25,26], when the IVC pressure exceeds the physiological threshold, directly leads to an increase in the mean right atrial pressure (RAP), causing mechanical distraction of the atrial wall (approximately 8 percent increase in atrial volume for every 1 mmHg increase in RAP) [27]. When the pressure gradient of the ventricular septum decreases from –3 mmHg to –5 mmHg in the end-diastolic phase, the left ventricular septum shifts to the end, resulting in a decrease in left ventricular end-diastolic volume and a significant increase in left atrial afterload [28,29], and this pressure overload environment promotes hypertrophy, disordered arrangement, and interstitial fibrosis of atrial myocytes, resulting in the formation of an electrically heterogeneous matrix [30,31]. Myocardial structural remodeling with abnormal ion channel function (e.g., accelerated L-shaped calcium channel inactivation) and shortened action potential duration (APD), increased ectopic triggering activity [32,33], and conduction block formation of reentrant circuits due to interstitial fibrosis form the pathophysiological basis for the maintenance of atrial fibrillation (Fig. 1) [34,35]

### 3.2 RAAS System Axis: The Molecular Link Between Venous Stasis and Atrial Fibrosis

Inferior venous blood stasis occurs when blood flow is impeded in the IVC, leading to obstruction of renal venous return (0.8 ± 0.3 mmHg for every 1 mmHg increase in IVC pressure) [36,37]. This obstruction triggers a decrease in effective perfusion pressure in the renal cortex, creating a hypoxic microenvironment in renal tissue [38]. Renal cortical hypoperfusion stimulates the secretion of renin by juxtaglomerular cells (JGC), resulting in a compensatory increase in plasma renin activity (*PRA*) [39]. Renin catalyses the conversion of angiotensinogen to angiotensin I (*Ang I*), which is transduced by angiotensin-converting enzyme (*ACE*) to produce angiotensin II (Ang II), resulting in a cascade amplification effect [40,41]. The increased secretion of *AngII* causes the following:

#### 3.2.1 Oxidative Stress Pathway

*AngII* activates the *NADPH* oxidase system through the angiotensin II type 1 (*AT1*) receptor, which acts as a core catalyst for reactive oxygen species (ROS) generation [42], and its increased activity directly promotes *ROS* production [43,44]. At the same time, *AngII* also increases mitochondrial superoxide anion (O_2_^–^) levels, inhibits superoxide dismutase (SOD) activity [45], and activates protein kinase C (*PKC*) isoenzymes (e.g., *PKCβ*), which enhance their activity by phosphorylating *NADPH* oxidase subunits [46,47], leading to ROS accumulation. In a cardiac electrophysiology study, *ROS* was found to modify cardiac ion channel proteins by oxidation, resulting in a transient decrease in the density of outward potassium currents and an acceleration of inward rectifying potassium current (*IK1*) inactivation [48], and these electrophysiological alterations significantly prolonged the APD time course and QT interval and provided an electrical matrix basis for atrial fibrillation triggering [49,50].

After Ang II activates the AT1 receptor, a significant amount of ROS is generated by NADPH oxidase (NOX). ROS can directly bind to nitric oxide (NO) to form peroxynitrite (ONOO⁻), which not only consumes NO but also leads to the “decoupling” of endothelial nitric oxide synthase (eNOS). This decoupling shifts eNOS from producing NO to producing ROS [51]. Additionally, Ang II inhibits NO production by downregulating the phosphorylation of eNOS (e.g., reducing phosphorylation at Ser1177) or by directly decreasing its protein expression [52]. Furthermore, Ang II activates the Ras homolog gene family, member A (RhoA)/Rho-associated coiled-coil–containing protein kinase (ROCK) pathway, which may inhibit eNOS activity [53], ultimately resulting in impaired endothelium-dependent vasodilation [54]. The reduced bioavailability of NO contributes to compromised endothelium-dependent diastolic function, leading to local tissue ischaemia and hypoxia [55]. In a study of nitric oxide and mechanical conduction in cardiomyocytes, it was found that NO not only regulates vascular tone but also affects ion flux (e.g., Na^+^, K^+^, or Ca^2+^) in cardiomyocytes through mechanosensitive channels (e.g., Piezo-type mechanosensitive ion channel component 1 (PIEZO1)) [56], and its abnormal signalling leads to shortened action potential duration and slowed conduction velocity, increasing the risk of atrial fibrillation [57].

#### 3.2.2 Abnormal Electrical Conduction Pathway

*AngII* binds to the *AT1* receptor and initiates the mitogen-activated protein kinase (*MAPK*)*/*extracellular signal-regulated kinase (*ERK*)*1/2* phosphorylation cascade, which influences the subcellular distribution and functional expression of Connexin 43 (*CX43*) by modulating its serine/tyrosine phosphorylation status [58]. A study examining the structure of the connexin-43 gap junction channel in its closed state revealed that *CX43*, as the core subunit of the myocardial cell gap junction channel, facilitates the exchange of intercellular ions (Ca^2+^, K^2+^) and metabolites (*ATP*, *cAMP*) through the formation of transmembrane channels [59]. This process ensures the synchronous conduction of electrical signals [60]. *AngII* induces phosphorylation modifications of *CX43* via the *MAPK/ERK1/2* pathway, promoting its internalisation from the cell membrane to the cytoplasm. The abnormal distribution of *CX43* significantly diminishes the electrical coupling efficiency between cardiomyocytes and increases the dispersion of repolarisation, leading to conduction directional disturbances and creating a local conduction velocity gradient (with longitudinal conduction velocity differences of up to 0.3 m/s). This provides an anatomical substrate for reentrant arrhythmias [61], and such complex heterogeneity can induce the formation of functional conduction block regions, thereby promoting the development of atrial fibrillation [62].

#### 3.2.3 Fibrosis Pathway

After *AngII* binds to the* AT1* receptor, it simultaneously activates the three major signalling axes of *MAPK* (including *ERK* and *JNK* subclasses),* PI3K/Akt*, and *TGF-β/Smad* to form a cascade response network [63]. In a study on the role of chemokines in epithelial-mesenchymal transition, it was proposed that the *TGF-β/Smad* pathway directly regulates the transcriptional activation of collagen genes (*COL1A1*, *COL3A1*) and fibronectin (*FN1*) by phosphorylating Smad2/3 by promoting the nuclear translocation β of the *Smad2/3-Smad4 *complex [64]. In addition, the *AngII*-activated* MAPK* pathway (e.g., *ERK*, *JNK*) forms a synergistic effect with *TGF-β/Smad* signalling [65], resulting in the differentiation of resting fibroblasts into myofibroblasts secreting the extracellular matrix (*ECM*), which produces large amounts of collagen (e.g., types I, III) and fibronectins, ultimately leading to interstitial fibrosis [66,67]. Myofibroblasts interfere with the electrical conduction of cardiomyocytes through electrotension coupling, forming an electrically heterogeneous fibrotic matrix [68]. Collagen deposition leads to segmentation of myocardial tissue, creating a nonconductive barrier that induces excitatory wave splitting and reentrant agitation, which induces atrial fibrillation [69].

*AngII* triggers a phosphorylation cascade within the downstream *MAPK* family through the *AT1* receptor. This cascade includes the activation of* p38 MAPK*, c-Jun N-terminal kinase (*JNK*), and *ERK* [70], which collectively promote cardiomyofibroblast proliferation by activating the *ERK1/2* and *JNK1/2* pathways [71]. Furthermore, a study on renal tubular epithelial protease-activated receptor 2 (*PAR2*) demonstrated that it promotes interstitial fibrosis by enhancing the inflammatory response and epithelial-to-mesenchymal transition (*EMT*) processes. Activated *MAPK* was found to induce the transition of epithelial cells to a mesenchymal phenotype by phosphorylating the *Snail/Slug* transcription factor. This transition is characterized by the upregulation of α-smooth muscle actin (*α-SMA*) and vimentin expression, along with increased *ECM* synthesis, resulting in a pro-fibrotic microenvironment [72]. The degree of myocardial interstitial fibrosis is positively correlated with electrical conduction heterogeneity, providing an anatomical basis for the development of atrial fibrillation. This occurs as the myocardial bundle is divided, forming a heterogeneous conduction matrix that leads to local conduction delays and disruptions, significantly increasing susceptibility to atrial fibrillation [69].

After *AngII* binds to the *AT1* receptor, it activates the G protein-coupled signal, triggers the translocation of *RhoA* from the cytoplasm to the membrane and binds to *GTP*, thereby activating the downstream *ROCK* kinase [73]. In a study on the effects of *RhoGTPase* and its downstream pathways on human aortic vascular smooth muscle cell (HA-VSMC) migration and phenotypic transformation, it was proposed that activated *ROCK* inhibits actin depolymerisation factor activity by phosphorylating LIM kinase (*LIMK*) and myosin light chain (*MLC*). This results in enhanced stability of F-actin microfilaments and the formation of stress fibres [74], while *ROCK* phosphorylates myosin phosphatase target subunit 1 (*MYPT1*), enhances myosin *ATPase* activity, and promotes the remodelling of the actomyosin contraction apparatus, a cytoskeletal remodeling that directly alters the morphology of atrial myocytes by increasing cell surface area and altering the longitudinal/transverse diameter ratio, resulting in a pathologically hypertrophic phenotype [75]. In addition, the *RhoA*/*ROCK* pathway, through the paracrine mechanism, promotes the transformation of fibroblasts into myofibroblasts, which secrete excess collagen to form a fibrotic microenvironment [76]. Sustained activation of this pathway results in a 20 to 40 percent increase in atrial myocyte surface area, accompanied by a disturbed distribution of connexins (e.g., *CX40*/*CX43*), resulting in increased conduction heterogeneity and a reentry matrix that induces the development of atrial fibrillation [77].

#### 3.2.4 Inflammatory Pathway

*AngII* binds to the *AT1* receptor and directly activates *NOX*, leading to the production of *ROS*. This process activates the *NF-κB* pathway by inhibiting the degradation of *IκB-α*, which drives the transcription of interleukin-6 (*IL-6*) and tumour necrosis factor-alpha (*TNF-α*) genes [78,79]. The *MAPK* and transforming growth factor-beta (*TGF-β*) pathways are coupled with the *AT1* receptor, creating a synergistic effect that further amplifies the inflammatory response mediated by *NF-κB* [80]. Several clinical studies have shown that plasma levels of *IL-6*, *TNF-α*, and matrix metalloproteinase-9 (MMP-9) are significantly elevated in patients with atrial fibrillation and are positively correlated with left atrial enlargement and fibrosis [81]. *IL-6* promotes the expression of fibrosis genes in cardiomyocytes through the *sIL*-*6R*/*STAT3* signalling axis [61], and *TNF-α* directly upregulates the expression of *MMP-1*, *MMP-3*, and *MMP-13* [82], and overexpression of these matrix metalloproteinases may lead to extracellular matrix degradation and atrial fibrosis, thereby promoting structural remodelling of the atria [83]. *IL-6* and *TNF-α* can also promote neutrophil and macrophage migration to atrial tissue by inducing the expression of chemokines (e.g., *CCL5 *and *CXCL10*), which secrete proteases such as *MMP-9* to directly degrade *ECM* components and release *ROS* and inflammatory factors to further activate fibroblasts to produce *MMP* [84]. An imbalance in the tissue inhibitor of metalloproteinase (TIMP)/*MMP* ratio (e.g., elevated *TIMP-1* or increased *MMP* activity) can lead to excessive collagen deposition, promote atrial fibrosis [85], and significantly increase the risk of atrial fibrillation [86].

#### 3.2.5 Calcium Homeostasis Imbalance Pathway

*AngII* enhances the secretion of presynaptic norepinephrine (*NE*) by activating the *AT1* receptor. This presynaptic effect directly promotes the release of sympathetic neurotransmitters, thereby increasing the activity of the sympathetic nervous system [87,88]. Additionally, *AngII* can prolong the duration of *NE* action in the synaptic cleft by inhibiting *NE* reuptake or metabolism, such as by reducing monoamine oxidase activity [89]. Norepinephrine activates the G protein (*Gαs*)-adenylyl cyclase (*AC*)*-cAMP* signaling cascade by binding to β-adrenergic receptors (*β-AR*) on the myocardial cell membrane, resulting in elevated *cAMP* levels and the activation of protein kinase A (*PKA*) [90]. *PKA* further exacerbates calcium overload by phosphorylating sarcoplasmic reticulum-associated proteins, which promotes calcium release and reuptake [91,92]. By increasing calcium influx, *PKA* activates calcium-calmodulin-dependent kinase II (*CaMKII*), which subsequently phosphorylates the calcium channel inhibitory protein Rad, creating a positive feedback loop that intensifies calcium overload [93]. However, increased calcium influx and the occurrence of calcium overload can shorten the effective refractory period of the atria [94], trigger ectopic electrical activity, and establish a critical substrate for atrial fibrillation [95], and increase the risk of atrial fibrillation [96].

#### 3.2.6 Prothrombotic State Pathway

**Fig. 2.**
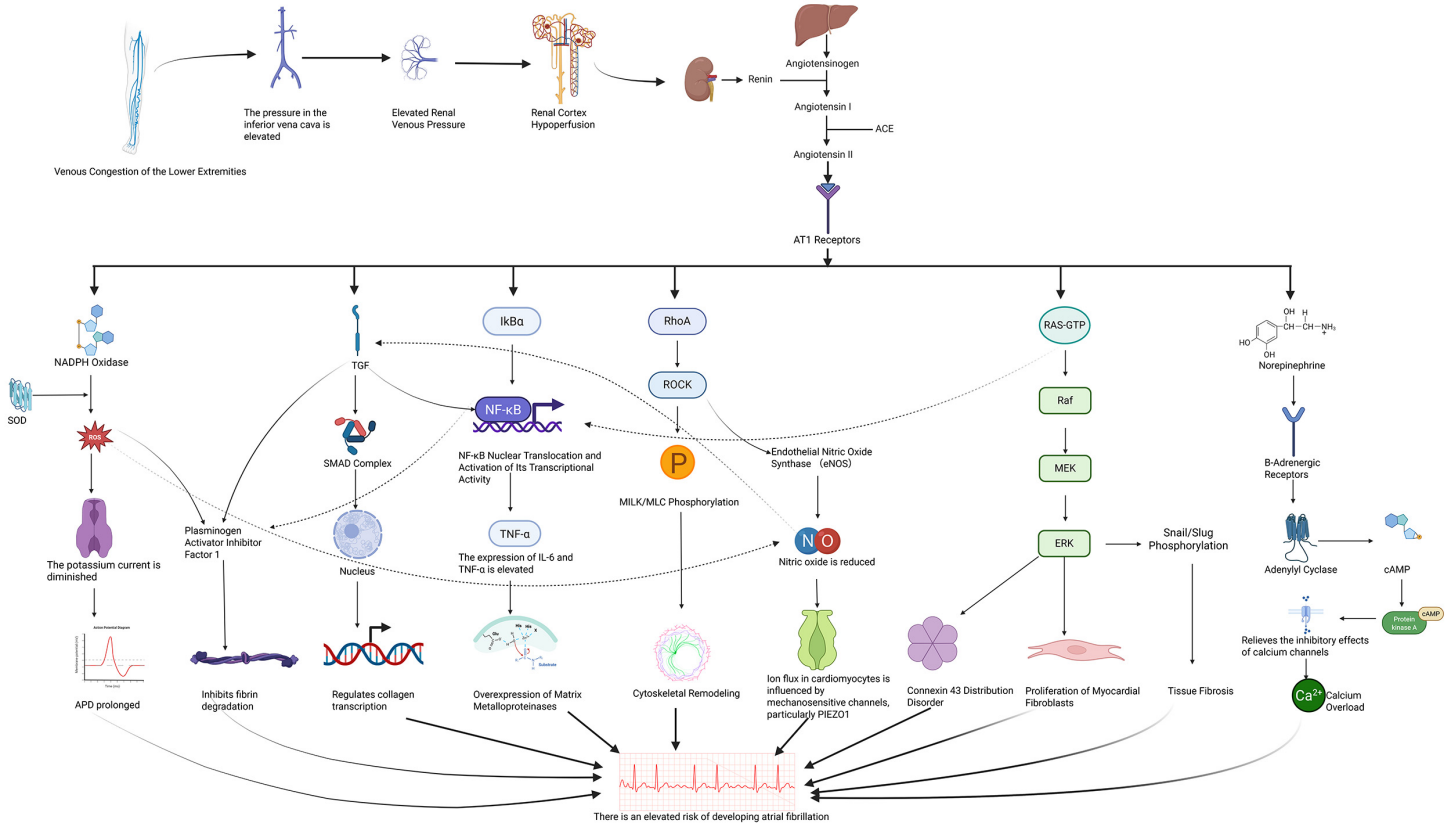
**RAAS system axis**. The figure was created using BioRender. ACE, angiotensin-converting enzyme; AT1, angiotensin II type 1; TNF-α, tumour necrosis factor-alpha; ERK, extracellular signal-regulated kinase; SOD, superoxide dismutase; APD, action potential duration; RAAS, renin-angiotensin-aldosterone system; MEK, mitogen-activated protein kinase kinase; ROCK, Rho-associated coiled-coil containing protein kinase.

*AngII* activates the *TGF-β1/Smad *pathway through the *AT1* receptor, upregulates the expression of plasminogen activator inhibitor 1 (*PAI-1*), and directly promotes the transcription and expression of *PAI-1* [97,98]. At the same time, *AngII* indirectly promotes the expression of* PAI-1* by stimulating inflammatory responses (e.g., *TNF-α, IL-6*, etc.) and oxidative stress (e.g., *ROS* production) [99], further activating *TGF-β* and Smad signalling pathways, forming a positive feedback regulatory network [76]. *PAI-1*, as a major inhibitor of the fibrinolytic system, significantly inhibits fibrin degradation by irreversibly binding tissue plasminogen activator (tPA) and urokinase-type plasminogen activator (*uPA*) to block the conversion of plasminogen to plasmin [100]. This process directly leads to impaired fibrinolytic function and an increased risk of thrombosis [100], and *PAI-1* also forms a stable complex with fibrin and *ECM* components (e.g., vitronectin), further prolonging fibrin retention and exacerbating thrombotic burden [100]. In a study exploring the effect of mitral regurgitation on the risk of thrombosis in patients with non-rheumatic atrial fibrillation, *PAI-1*-mediated hypercoagulability was found to lead to atrial electrical remodelling through oxidative stress and the calmodulin signalling pathway, resulting in an electrophysiological matrix for the development of atrial fibrillation [101] (Fig. 2).

### 3.3 Sympathetic Storm: The Vicious Cycle of Autonomic Nervous System Remodeling

In saphenous varices, distal venous pressure overload results from a deficit in venous return from the lower extremities [102]. Fluctuations in CVP are indirectly caused by increased resistance to venous return or changes in venous volume [103]. Elevated central venous pressure alters the sensitivity of baroreceptors to arterial pressure, resulting in a rightward shift of the pressure-volume curve [104]. This triggers a chronic reset of the baroreflex through long-term activation of the venous dilation reflex [105], which leads to sustained sympathetic excitation, exacerbating vascular dysfunction and impairing blood pressure regulation [106]. Increased sympathetic activity may shorten the effective refractory period of the atria [107] by enhancing calcium currents or reducing repolarisation time through the activation of β-adrenergic receptors. This results in a shortened atrial action potential time course and a decrease in conduction wavelengths, thereby shortening the effective refractory period and promoting the development of reentrant arrhythmias [108,109]. Consequently, both sympathetic hyperactivity and relative inhibition of parasympathetic function contribute to a shortened time course of atrial action potentials and a reduction in conduction wavelengths, thereby facilitating the formation of a reentry mechanism [109] and significantly increasing susceptibility to atrial fibrillation [107].

### 3.4 Abnormal Shear Stress: The Bidirectional Communication System of the Vascular Endothelium

**Fig. 3.**
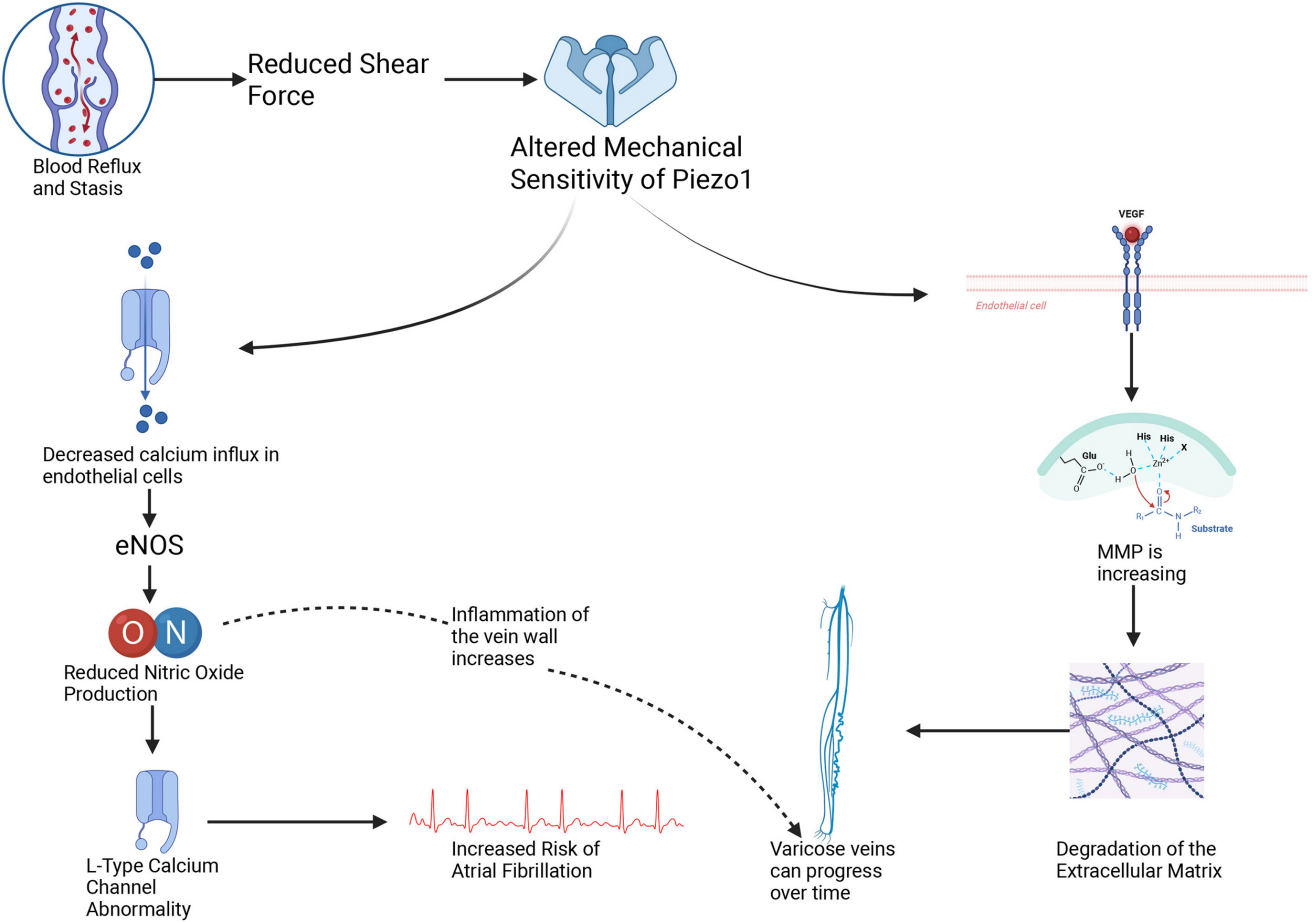
**Abnormal shear forces cause bidirectional signalling imbalances in the endothelium**. The figure was created using BioRender. eNOS, endothelial nitric oxide synthase; MMP, matrix metalloproteinase.

Venous valve insufficiency in patients with saphenous varices leads to blood reflux and stasis, which reduces blood flow velocity and consequently diminishes shear forces [110]. The veins themselves exist in a low-shear environment, further exacerbated by flow disturbances in pathological states [111]. Research indicates that low shear forces alter the mechanosensitivity of the Piezo1 ion channel through specific integrins (e.g., *αvβ3/αvβ5*) [112] and directly decrease the opening probability of Piezo1 by diminishing mechanical stimulation [113]. This suggests that reduced blood flow shear forces can result in the inactivation or functional inhibition of the endothelial Piezo1 mechanosensor via multiple molecular pathways. Studies have shown that endothelial cells upregulate* MMP-9* through the *VEGF* or *CXCR4* pathways when the shear-sensitive ion channel Piezo1 mechanosensor is inactivated [114], and these enzymes further promote venous wall weakness and varicose progression by degrading the *ECM* and affecting the endothelial, smooth muscle, and matrix components of the venous wall [115,116]. This in turn affects downstream signalling pathways (e.g., *Orai1*, *CaMKII*, *CXCR4*, etc.), resulting in abnormal vascular function and dysregulated inflammatory response [117,118]. Activation of *eNOS* requires calcium ions to bind to calmodulin, and decreased calcium influx leads to an inhibition of this process [119], and calcium influx restriction also affects mitochondrial *ATP* production, indirectly inhibiting *eNOS* activity [120], resulting in reduced *NO* production. Decreased *NO* synthesis can exacerbate venous wall inflammation by weakening the anti-inflammatory capacity of endothelial cells and promoting the release of pro-inflammatory factors (e.g., *IL-6*) [121,122], which exacerbates the progression of varicose veins. On the other hand, decreased *NO* synthesis is associated with L-type calcium channel dysfunction [123,124], and L-type calcium channel dysfunction in atrial fibrillation atrial myocytes is one of the core mechanisms of electrical remodelling, so endothelial dysfunction (e.g., decreased *NO*) is also associated with an increased risk of AF [125] (Fig. 3).

### 3.5 Circulating Microparticles: Nanoscopic Messengers for Inter-Organ Communication

Endothelial dysfunction in patients with varicose veins is characterised by elevated levels of endothelial cell-derived microparticles (*EMPs*, *CD144*) that may enter the systemic circulation through the bloodstream [126]. These systemic microparticles can return to the right atrium via venous reflux, thereby affecting atrial tissue function [127]. Studies have demonstrated that increased circulating levels of *EMPs* (as indicated by the *CD144* marker) in patients with atrial fibrillation may promote atrial thrombosis and embolic events through procoagulant activity and endothelial dysfunction. This hypercoagulable state may indirectly lead to atrial electrical remodelling or structural abnormalities that induce atrial fibrillation [128]. Additionally, these particles carry bioactive molecules (e.g., microRNAs and inflammatory factors) that act on atrial tissues through paracrine or circulatory pathways, promoting fibrosis, inflammation, and electrical remodelling. Eventually, atrial fibrillation develops [127,129]. *EMP* may damage the atrial endothelial barrier through oxidative stress pathways and activate the *ERK*/*MAPK* and *NF-κB* signalling pathways, resulting in increased secretion of inflammatory factors such as *IL-6* and *IL-1β* [130], exacerbating the inflammatory response [131], and ultimately leading to inflammatory cell infiltration and atrial tissue damage, thereby inducing atrial fibrillation [132]. However, the existing literature has not clearly established a direct causal chain between varices, endothelial particles, and atrial tissue, and the relevant mechanism still needs to be further experimentally verified [127].

## 4. Conclusion

This study systematically developed a pathophysiological framework linking atrial fibrillation to dysfunction of the great saphenous vein, elucidating the key mechanisms by which lower limb venous lesions contribute to atrial electrical and structural remodelling through multiple pathways. This novel approach connects venous abnormalities in the legs to atrial fibrillation, extending beyond traditional studies that focus on a single organ, and offers a new perspective on how leg vein pathology can lead to atrial fibrillation. Furthermore, it provides new insights for follow-up research and clinical practice, serving as a reference to enhance atrial fibrillation screening in patients with varicose veins.
